# Physiological Recordings of the Cerebellum in Movement Disorders

**DOI:** 10.1007/s12311-022-01473-6

**Published:** 2022-09-07

**Authors:** Ami Kumar, Chih-Chun Lin, Sheng-Han Kuo, Ming-Kai Pan

**Affiliations:** 1 Department of Neurology, Columbia University Irving Medical Center and the New York Presbyterian Hospital, 650 W 168thStreet, Room 305, New York, NY 10032, USA; 2 Initiative for Columbia Ataxia and Tremor, Columbia University Irving Medical Center, New York, NY, USA; 3 Cerebellar Research Center, National Taiwan University Hospital, Yun-Lin Branch, Yun-Lin 64041, Taiwan; 4 Department and Graduate Institute of Pharmacology, National Taiwan University College of Medicine, Taipei 10051, Taiwan; 5 Department of Medical Research, National Taiwan University Hospital, Taipei 10002, Taiwan; 6 Institute of Biomedical Sciences, Academia Sinica, Taipei City 11529, Taiwan

**Keywords:** Cerebellum physiology, EEG, MEG, Ataxia, Essential tremor, Dystonia

## Abstract

The cerebellum plays an important role in movement disorders, specifically in symptoms of ataxia, tremor, and dystonia. Understanding the physiological signals of the cerebellum contributes to insights into the pathophysiology of these movement disorders and holds promise in advancing therapeutic development. Non-invasive techniques such as electroencephalogram and magnetoencephalogram can record neural signals with high temporal resolution at the millisecond level, which is uniquely suitable to interrogate cerebellar physiology. These techniques have recently been implemented to study cerebellar physiology in healthy subjects as well as individuals with movement disorders. In the present review, we focus on the current understanding of cerebellar physiology using these techniques to study movement disorders.

## Introduction

Located in the posterior fossa, the cerebellum, also known as the “little brain,” is a brain structure important for controlling the precision of movements. The cerebellum has functional connectivity with other parts of the brain and is highly integrated into the network with the cerebral cortex, brainstem, and spinal cord. However, how the cerebellum modulates voluntary movements and how the dysfunctional cerebellum causes movement disorders remain important research topics and have implications in developing neuro-modulatory strategies. Along these lines, cerebellar physiological recordings in healthy individuals and/or in patients with a variety of movement disorders have recently gained interest in neuroscience research.

The cerebellum has unique anatomical and physiological attributes. The cerebellum contains 10 different lobules and can be further divided into vermis, paravermis, and hemispheres. The cerebellum is also a highly folded structure with many folias, and the surface of the cerebellum is estimated to be 80% of the surface area of the cerebrum [1]. The principal neurons of the cerebellum are Purkinje cells, which have firing frequencies of 50–140 Hz. Purkinje cell activity is tightly controlled by the two excitatory synaptic inputs: climbing fibers from the inferior olivary neurons and parallel fibers from the granule cells [2]. In addition, inhibitory inputs from the interneurons and also the intrinsic pace-making activity of Purkinje cells modulate Purkinje cell firing patterns.

Cerebellar physiological signals were originally described by direct and invasive intracranial electrodes. There were four studies published, in which three were in Russian and one was in French [3–6]. These studies describe cerebellar recordings with oscillatory activity of high frequencies, in the range of 220–250 Hz. These invasive investigations have given important insights into cerebellar physiology and function.

While intracranial cerebellar recordings are important techniques, such precision is not yet possible to be widely adopted to study cerebellar physiology in patients. Instead, electroencephalogram (EEG) or magnetoencephalogram (MEG) that presumably detect synaptic activity integration are often used to study cerebellar physiology in humans. There are various studies that have used the application of EEG and MEG, in the domains of motor, auditory, visual, and cognition in the cerebral cortex in association with the cerebellum. These studies mostly involve healthy subjects and patients with epilepsy and have recently been reviewed [7]. We will instead focus on the cerebellar physiological features in movement disorders.

Cerebellar dysfunction has been implicated in three main categories of movement disorder phenomenology: ataxia, dystonia, and tremor. What is the cerebellar physiology in these diseased states and what are the technologies and analyzing methods used to study cerebellar physiology in diseases? We aim to address these questions in this review. We will first review the clinical characteristics of each movement disorders phenomenology and the evidence of cerebellar involvement, including animal studies. We will then review physiological studies of the cerebellum in patients with these movement disorders to provide a context of the current state of the field and also future research directions.

### Cerebellar Involvement in Ataxia, Tremor, and Dystonia

Three types of movement disorders phenomenology are related to cerebellar dysfunction: ataxia, tremor, and dystonia. The key clinical feature of ataxia is incoordination, imbalance, and irregular movements. On the other hand, tremor is characterized by rhythmic and oscillatory movements. Dystonia is involuntary, sustained muscle contractions, leading to abnormal repetitive movements and postures. These three types of abnormal movements can be highly disabling to patients and therapeutic options are rather limited. Therefore, understanding how the dysfunction of the cerebellum can cause these unique movements will help to develop targeted treatments for patients suffering from ataxia, tremor, and dystonia. As ataxia, tremor, and dystonia are movement disorders symptoms, we will next introduce the prototypical neurological disorders associated with ataxia, tremor, or dystonia because these disorders are often the subject for studying the cerebellar physiological correlates with each movement disorders phenomenology.

Ataxia is classically associated with cerebellar degeneration. The prototypical diseases of ataxia are spinocerebellar ataxia (SCA), which are caused by genetic mutations. Other ataxic disorders include multiple system atrophy cerebellar type (MSA-C) and idiopathic late onset cerebellar ataxia (ILOCA), which are late onset, degenerative causes [8]. Friedreich’s ataxia (FRDA) is the most common childhood onset ataxic disorder. While FRDA is traditionally thought to be related to sensory neuronopathy, cerebellar involvement, specifically the dentate nucleus degeneration is evident in FRDA [9]. In the pathological examination of post-mortem cerebellum in patients with ataxia such as SCA and MSA-C, loss of Purkinje cells and other associated degenerative changes are common [10]. In neuroimaging findings, cerebellar atrophy is the hallmark feature of ataxic disorders [8].

Tremor is another hyperkinetic movement found to be associated with cerebellar dysfunction. The prototypical disease for tremor is essential tremor (ET), which is the most common movement disorder [11]. The second most common tremor disorder is parkinsonian tremor (i.e., tremor in Parkinson’s disease (PD)). ET is characterized by action tremor whereas parkinsonian tremor is rest tremor. Both types of tremor can be effectively suppressed by deep brain stimulations to the thalamus that received cerebellar outflows [12], providing convincing evidence of cerebellar involvement in these tremor disorders. Other tremor disorders include orthostatic tremor, which manifests as leg tremor during standing, and tremor associated with degenerative causes such as Wilson’s disease. Multiple lines of neuroimaging studies have also demonstrated that the cerebellum is a critical brain region in ET and parkinsonian tremor [13–17]. In the post-mortem pathological examination, ET brains have some cerebellar degenerative changes, including modest Purkinje cell loss [18, 19]. In addition, abnormal synaptic connections of climbing fibers and Purkinje cells have been identified in the ET cerebellum [20, 21], indicating cerebellar dysfunction. How does the dysfunctional cerebellum create tremor? In the harmaline-induced tremor models, Purkinje cells are driven by synchronized olivary neuronal inputs to have synchronous and rhythmic activity, suggesting that synchronized and rhythmic neuronal activity in the cerebellum could be the physiological underpinning of tremor [22]. In PD patients, alpha synuclein associated pathological changes have been linked to tremor symptoms [23–25]. Alpha synuclein formed Lewy bodies have been found in the Bergmann glia and Purkinje cell axons in the cerebellum. Also, in PD patients, there is a presence of increased climbing fiber-Purkinje cell synapses as well as the presence of torpedoes in Purkinje cell axons demonstrating cerebellar involvement in PD [26, 27].

Dystonia is the third hyperkinetic movement related to the cerebellum. Different from ataxia and tremor, both the basal ganglia and the cerebellum play roles in dystonia. Emerging evidence demonstrates that the cerebellum may be a predominant player in certain forms of dystonia [28, 29]. The prototypical disorders of cerebellar-linked dystonia are genetic forms, including DYT1, DYT11, and DYT12 dystonia [30–32], which may present as focal, segmental, or generalized dystonia. Writer’s cramp is a common form of idiopathic, focal dystonia, which involves involuntary muscle contractions causing difficulty in writing. Neuroimaging studies in dystonia have shown evidence of cerebellar involvement [33].

### Encephalographic Techniques for Recording

As mentioned above, invasive cerebellar recordings in humans are exceptionally rare and have not been widely implemented in studying human movement disorders. On the other hand, emerging evidence demonstrated that network analysis using EEG and MEG in humans can detect the synchronization of the cerebellar activity, either by itself or with the cerebral cortex. These techniques allow us to probe the role of the cerebellum in movement disorders.

**Fig. 1:**
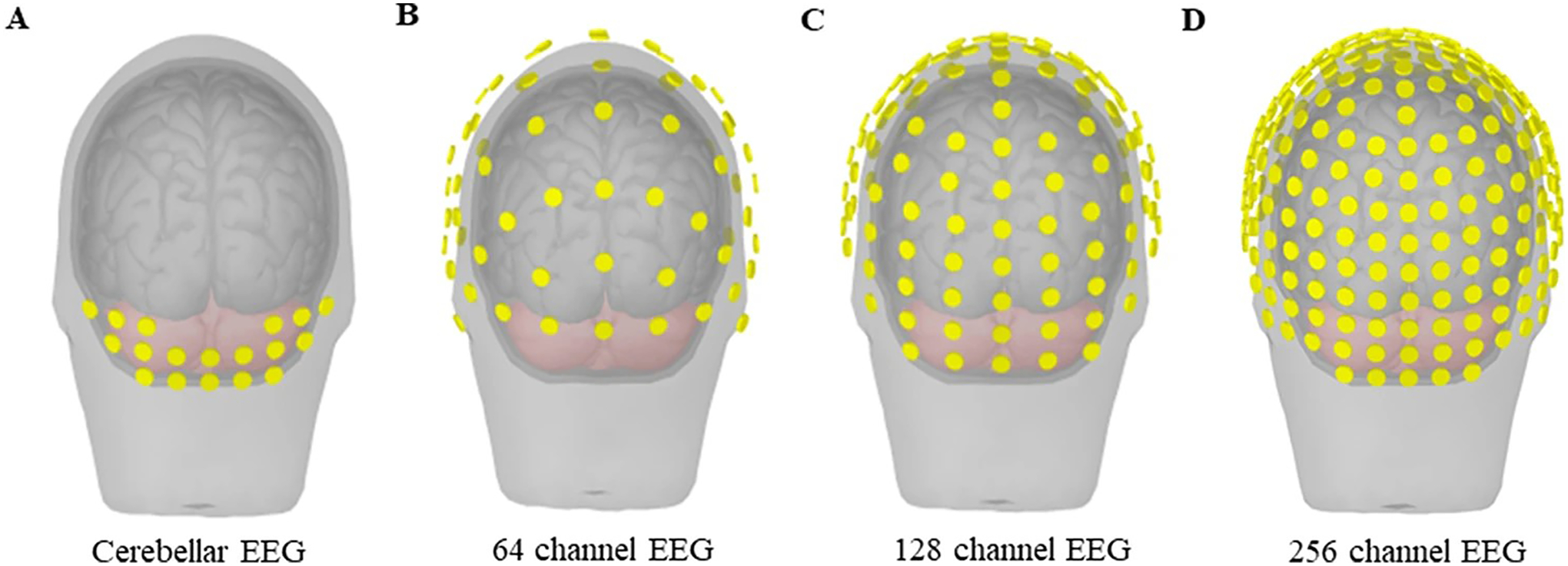
Schematic representation of EEG channel placement. Cerebellar coverage based on the different EEG channel layouts. (**A**) Cerebellar EEG layout. (**B**) 64-channel EEG layout. (**C**) 128-channel EEG layout. (**D**) 256-channel EEG layout

EEG is a technique by which the electrical activity of the human brain can be measured from the scalp noninvasively. Based on the study focus, the number of EEG channels and locations may differ in order to record cortical or cerebellar activity. Figure 1 provides a schematic diagram of EEG, with different channel montages covering the cerebellar area. EEG signals can be further processed by dividing the signals into different frequency bands and the underlying neural correlates can be associated with certain behavioral and cognitive processes [34, 35], and in our case, with specific movement disorders symptoms. EEG is also often used in the analyses of event related potentials, which is extensively used in studying PD [36, 37]. EEG is a powerful tool to study physiological connectivity, by investigating the association of signals recorded from different brain regions and the disruption of such connectivity in diseased states [38, 39]. Different from EEG, MEG is a technique that records brain activity based on the magnetic fields generated by electrical currents in the brain. EEG and MEG can detect signals from the surface of the brain, such as the cerebral and cerebellar cortex, as well as further probe deeper brain structures, such as the basal ganglia and thalamus [40–43].

While EEG and MEG have an excellent temporal resolution of brain activity, the major limitation of such techniques is low spatial resolution, posing a challenge to precisely identify the sub-domain of a given brain structure; for example, the specific lobule of the cerebellum. Nevertheless, over the past few years, an increasing number of studies have demonstrated that detecting cerebellar signals using EEG and MEG is indeed possible [7, 44]. EEG has an excellent temporal resolution, which can complement the limitation of functional neuroimaging studies. We will review cerebellar recording in each of the movement disorders phenomenology in detail.

## Methods for Literature Search

**Fig. 2:**
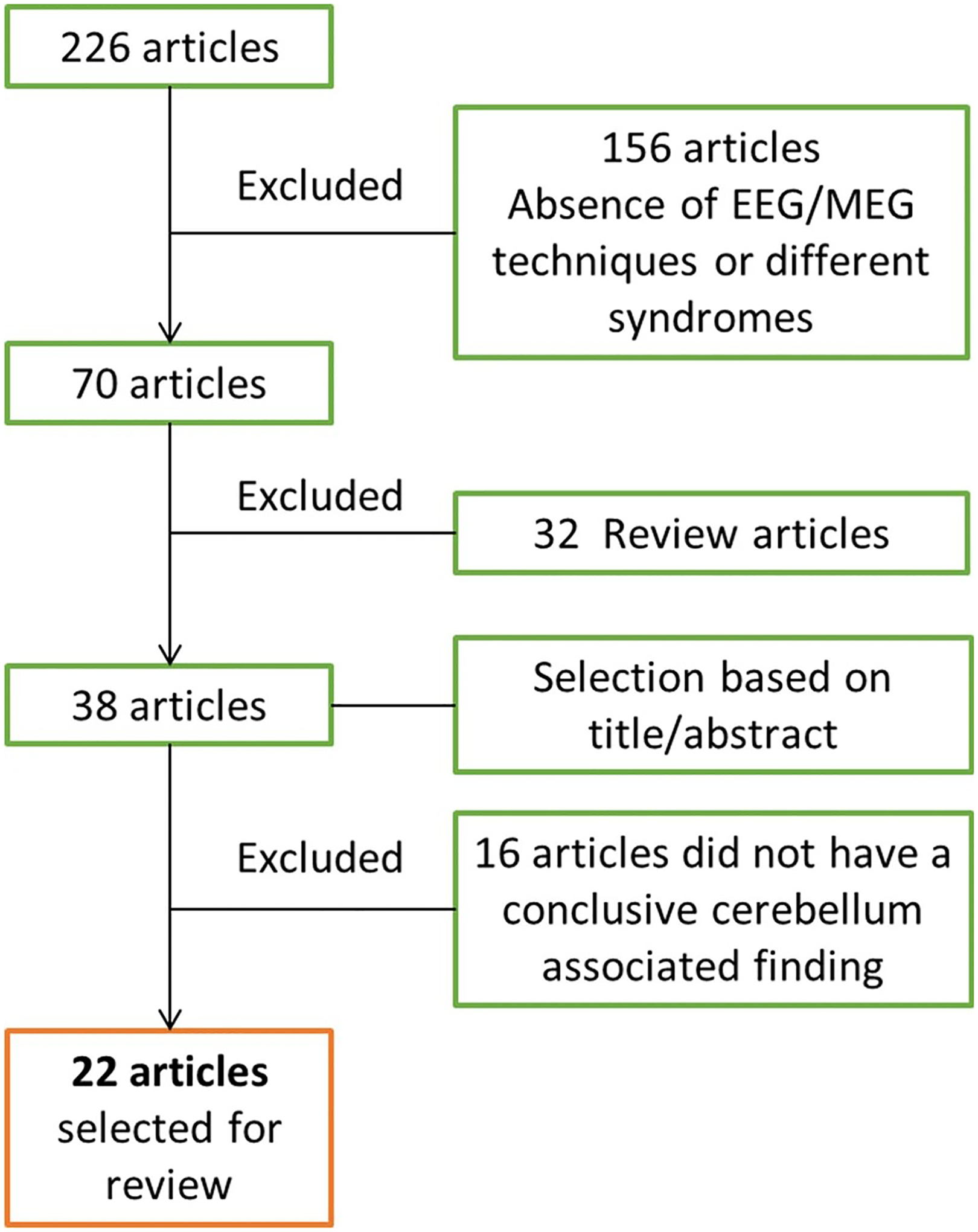
Schematic representation of article selection. Selection criterion for articles in the present review, consisting of studies using EEG or MEG as techniques and having findings linked to cerebellar physiology in ataxia, tremor, and dystonia

We performed a PubMed search using the key words, cerebellum, EEG, MEG, tremor, ET, PD, ataxia, and dystonia. Additionally, filters for human research and the English language were applied. Articles were included in the review if the study methods were EEG or MEG techniques, and the results include cerebellum associated findings for ataxia, tremor, or dystonia. The search generated 226 articles. We excluded 156 articles because either these studies did not use EEG or MEG, or did not study ataxia, tremor, or dystonia. Furthermore, we excluded 32 review articles. Based on the title and the abstract, 38 articles were selected. Sixteen of these articles were further excluded, as these articles did not have any cerebellum associated findings. The remaining 22 articles were selected for this review since these studies fulfilled the inclusion criteria (Fig. 2). Among the 22 studies, 6 studies are on EEG studies of the functional brain network in patients with cerebellar ataxia without direct cerebellar physiology measurement, which help us to understand the brain activity in responses to cerebellar degeneration. Given the paucity of cerebellar physiological studies for ataxia patients (*n* = 2), we thus included these additional 6 studies.

## Cerebellar Physiology in Ataxia

In ataxic disorders, cerebellar physiology recording by EEG or MEG is often done in the context of whole brain recording, and the data is analyzed using source localization and functional connectivity analysis (summarized in Table 1). In a MEG-based resting state functional connectivity study, Naeije et al. investigated determinants of FRDA associated changes in intrinsic functional brain network in 18 FRDA patients. The resting state functional connectivity was estimated, using 37 nodes or points of interest from the cerebellum and 6 major resting state networks: default mode, sensory motor, language, visual, ventral, and dorsal attention networks. In addition, they found that the cerebellar network is one of the major networks to be associated with the age of onset in FRDA, demonstrating that cerebellar physiology could be a predictor of disease course. Another finding in the study is that a higher resting state functional connectivity was linked to later onset of symptoms, supporting that this technique may be used to study the compensatory mechanism [45]. Song et al. investigated cerebello-frontal connectivity dynamics in patients with MSA-C, to test the target engagement of 10-day, intermittent theta burst stimulations by repetitive transcranial magnetic stimulation (rTMS) to the cerebellum. Twenty-five patients with MSA-C received rTMS stimulations whereas another twenty-five patients with MSA-C received *sham* stimulations in this randomized, double-blinded clinical trial. In the rTMS group, not only did these stimulations improve ataxia symptoms, as measured by the scale of ataxia rating and assessment (SARA), there was significantly increased cerebello-frontal connectivity, measured by time-varying EEG network patterns. On the other hand, the *sham* stimulated group did not have any significant clinical improvement or cerebello-frontal connectivity changes [46]. These studies provide the rationale that network analysis using EEG recording over the cerebellar region could be useful in predicting the disease onset and/ or serve as a biomarker for ataxic clinical trials.

As the brain function as a network, investigators also studied EEG signals in the cerebral cortex in ataxic patients to understand the network changes in response to cerebellar degeneration. While these studies do not directly measure cerebellar physiology, they provide a broader understanding of brain network changes in ataxic patients; therefore, we also included these studies in Table 1. Peterburs et al. investigated the role of the cerebellum in saccadic performance monitoring which measures simultaneous monitoring and evaluation of actions, using EEG in ataxia patients of various diagnoses. An antisaccadic task was performed during EEG recording, and ataxia patients were found to have increased error rates alongside reduced error-related negativity (ERN). There was also a reduction in the event related potential correlated with error processing and performance monitoring. However, error positivity was seen to be preserved in patients. This study demonstrated that patients with cerebellar ataxia have task-specific EEG signal alterations [47].Another approach to better understanding physiological aspects of ataxia is to assess motor tasks using event related potentials or event related synchronization (ERS)/event related desynchronization (ERD) analysis. Li et al. studied event related potentials in 12 SCA patients and 12 healthy controls with EEG. They investigated the role of the cerebellum in feedback control since the cerebellum plays an important part in the feed forward control of speech production. Behavior analysis revealed that patients with SCA have a significant increase in vocal compensations for pitch perturbations, which corresponds to a reduction of cortical activity in temporal-parietal regions, localized by source analysis. These results suggested that cerebellar degeneration affects the feedback control of speech production through the temporal-parietal regions. The cerebellum is critical for feed-forward control and feedback-based control for speech production [48]. Verleger et al. observed event-related potentials using EEG in 12 patients with cerebellar ataxia. Motor tasks such as coordination and control tasks were performed. Ataxic patients had impaired motor task performance and reductions in cerebral cortical EEG potentials during preparation and execution of movements when compared to controls. However, controls and ataxic patients had a similarly increased cortical EEG potential activity, when performing movements requiring higher level coordination. The results of this study depict that the cerebellum is mostly involved in the preparatory and executive activity, while for fine coordination, the motor cortex plays an active role [49]. Additionally, in a study by Yamaguchi et al., the event-related evoked potentials and reaction times in ataxic patients with cerebellar degeneration were investigated to understand the role of cerebellum in visuospatial attention shift. In ataxic patients as compared to controls, the effect of cerebellar degeneration was observed on event related evoked potentials by a reduced late negative deflection. This reflects that neural activities in correlation to preparation and selection for response are reduced. The study shows that the lateral cerebellum has a reduced involvement in the mechanism of visuospatial attention shift. However, the essential role of the lateral cerebellum is observed in distributed motor control systems [50].

As for studies involving ERD/ERS analysis, Visani et al. assessed patients with SCA based on EEG rhythms during a Go/No-go motor task. They found that SCA patients had a defective ERD lateralization in the cortical EEG recording. These defects in alpha and beta ERD/ERS analysis suggested impaired cerebellar modulation of the motor cortex [51]. In another ERD/ERS analysis on SCA3 patients using EEG and EMG, Aoh et al. observed decreases in ERS for frequencies 20–30 Hz, and further focused on the important role of the cerebellum in motor termination [52]. Collectively, these studies provide evidence of cerebellar modulation of cortical excitability.

## Cerebellar Physiology in Tremor

Various studies are using the approach of source localization and coherence analysis based on physiological data obtained using EEG and MEG to understand the underlying neuronal networks in tremor (summarized in Table 2). Muthuraman et al. conducted a study in 2015, on early onset ET and aging-related tremor patients using whole brain EEG, which also covers the cerebellar area. Simple motor tasks involving pinch grip task, holding task, and slow hand movements were conducted during which the pooled coherence spectra were estimated. Moreover, this study also used dynamic imaging of coherent sources (DICS) and renormalized partial directed coherence (RPDC) for source analysis and network analysis. They found that early onset ET patients have strong cerebellar involvement whereas age-related tremor patients do not, supporting that these analytical methods of EEG can be used to study cerebellar physiology of tremor [53]. In another MEG study of ET using DICS, Schnitzler et al. found that the brain network for tremor involves the primary motor cortex, premotor cortex, thalamus, cerebellum, and brainstem [54]. This study provides an important understanding that the interactions of these brain areas, including the cerebellum, can be critical for tremor generation in ET. Timmerman et al. studied the oscillatory brain network in parkinsonian rest tremor. Using DICS, this MEG study describes the cerebro-muscular, cerebro-cerebral, and partial coherence within cerebral areas and muscles. Notable findings in this study are the impaired coupling in a cerebello-diencephalic-cortical network, implying abnormal interactions of the cerebellum and other brain regions are the contributing factors to parkinsonian tremor [55]. Pollock et al. described the effects of levodopa on the oscillatory networks associated with parkinsonian rest tremor. Ten PD patients and 11 healthy controls were studied using MEG and EMG techniques. PD patients had an overnight withdrawal of levodopa and after oral intake of fast-acting levodopa to study the brain signals in the off and on periods. The oscillatory network of tremor at 8–10 Hz, as revealed by DICS, consisted of these brain regions: contralateral primary sensorimotor cortex, supplementary motor area, contralateral premotor cortex, thalamus, secondary somatosensory cortex, posterior parietal cortex, and ipsilateral cerebellum. Further, decreases in the coupling between thalamus and motor cortical areas were observed following levodopa intake [56].

In a study done in 2012, Muthuraman et al. described the differences associated with oscillatory network dynamics in parkinsonian tremor, ET, and voluntary movements. The study used DICS and EEG-EMG based coherence, in 10 PD patients and 10 ET patients. The network associated with basic tremor frequency consisted of the primary sensory motor cortex, prefrontal/premotor cortex, and anterior diencephalon (thalamus). The study further revealed for the selective time interval with high coherence, the source in the cerebellum was identified for all ET, PD, and healthy subjects, while ET patients had an additional brainstem source. These sources in the study were observed only during phases of high signal to noise ratio, due to limited electrodes in the posterior region. For voluntary tremor, a unidirectional flow of information was seen from the diencephalon to the cortex. On the other hand, PD and ET patients had bidirectional subcortico-cortical flow. The findings of this study help us to understand that both pathological tremor and voluntary mimicked tremor have similar brain network, demonstrating that both voluntary and pathological tremors involve the cerebellum, but the additional brain regions involved and the hierarchical order of the brain network may differ [57]. Additionally, in 2018, Muthuraman et al. used high-density 256 channel EEG coupled with EMG to study the cerebello-thalamo-cortical network in patients with PD, ET, and healthy controls (mimicked tremor). The coherence maps were constructed, and effective connectivity was established based on the partial directed coherence. They found that parkinsonian tremor and ET have the functional connection between the sensorimotor cortex and the cerebellum, while mimicked tremor in healthy subjects has the functional connection between premotor cortex and the cerebellum. Moreover, the oscillations flow from EMG to the cerebellum in mimicked tremor, while the oscillations flow from the cerebellum to EMG in parkinsonian tremor and ET. The results suggest altered cerebellar mechanisms involved in these three types of tremor, and the cerebello-cortical loop is important for parkinsonian tremor and ET [58]. Pedrosa et al. investigated the network difference associated with ET and voluntary tremor, in relation to alcohol intake, using high-density EEG and subject specific structural MRI in 20 ET patients and 20 age-matched controls. Cortical tremor coherence was observed distinctly in sensorimotor cortices for control subjects; however, for ET patients, the involvement of supplementary motor area was more prominent. The intake of alcohol was associated with reduced tremor amplitude and further linked with changes in cerebellar activity [59].

In a 2013-based source and coherence study, Muthuraman et al. investigated the central oscillatory network associated with orthostatic tremor. The corticomuscular coherence was analyzed using EEG and EMG recordings from 15 patients. The standing tremor network comprises of unilateral activations in primary motor leg area, supplementary motor area, primary sensory cortex, prefrontal/premotor area, thalamus, and cerebellum. On analysis of tremor network over time, the sources in the primary leg area and the thalamus with the tibialis anterior muscle were highly coherent for 30 s contralaterally, followed by decreases ipsilaterally after 15 s. The corticomuscular coherence pattern in orthostatic tremor is initially bilateral followed by a segregated unilateral pattern [60]. Finally, Südmeyer et al. investigated the underlying brain networks in association with the primary sensorimotor cortex in 5 Wilson’s disease patients with postural tremor, using MEG and EMG recordings with DICS. The oscillatory network is comprised of significantly coherent cortical and subcortical brain regions: primary sensorimotor cortex, premotor cortex, supplementary motor area, posterior parietal cortex, contralateral thalamus, and ipsilateral cerebellum. The findings of this study demonstrate the involvement of a cerebello-thalamo-cortical network in postural tremor in Wilson’s disease [61].

The above studies use the network and coherent analysis to study the interaction of the cerebellum and other parts of the brain in tremor. The next question is whether abnormal physiological signals can be directly recorded from the cerebellar region using EEG, without coherence analysis with other brain areas? As one of the latest techniques, our group, Pan et al. used EEG over the cerebellar region (cerebellar EEG) as a means to investigate and validate the contributing factors for tremor pathophysiology based on the identification of the synaptic pruning deficits of cerebellar climbing fibers in the postmortem ET brain. Consistently, we found that ET patients have ~ 10 Hz oscillatory activity in the cerebellum region, which was not seen in age-matched healthy controls. Interestingly, the strength of such cerebellar oscillatory activity correlates with tremor severity [62], as the first cerebellar physiological study in tremor with clinical-physiological correlates of disease severity. As a follow-up study, our group, Wong et al., also used cerebellar EEG to investigate cerebellar physiology in familial and sporadic ET patients, since about 43–63% of ET patients reported a family history of tremor [63–65]. Familial ET and sporadic ET may be considered as different ET subtypes and thus may have different underlying cerebellar physiology [66]. Interestingly, we found that both familial and sporadic ET patients have excessive cerebellar oscillatory activity, indicating that this abnormal EEG signal from the cerebellum is conserved in a heterogeneous ET population. Furthermore, it was observed that cerebellar oscillation in familial ET correlated with tremor severity, but this was not observed in sporadic ET [67].

Additionally, cerebellar physiology has also been explored in PD, outside of tremor, and we describe them here to provide additional context. Bosch et al. investigated the resting state cerebellar oscillations in 75 PD and 39 healthy controls, using cerebellar EEG. Their study describes finding increased theta frequency band (4–7 Hz) activity in cerebellar electrodes for PD patients as compared to controls. The findings of this study demonstrate that the abnormal theta band cerebellar oscillations from the cerebellum may play a role in the pathophysiology of PD [68]. Furthermore, Bosch et al. investigated cortical and cerebellar oscillatory responses using EEG in 10 PD patients with postural instability, 11 without postural instability, and 15 controls. Low theta band activity was observed in the mid-frontal and mid-cerebellar regions for PD patients with postural instability as compared to PD patients without postural instability, and controls. [69]. In another study, Bosch et al. investigated cerebellar EEG during cognitive and motor tasks. For this study, 79 PD patients and 37 controls were recruited, and cognition was evaluated during an interval timing task, while motor activity was evaluated using a lower limb pedaling task. Connectivity analysis between the mid-frontal and mid-cerebellar regions depicted differences in theta band during the motor task but not the cognitive task. As compared to controls, PD patients demonstrated altered cerebellar oscillations in the cognitive and motor tasks [70].

## Cerebellar Physiology in Dystonia

Several studies have described the neurophysiological aspects of dystonia, involving the cerebellum, based on non-invasive techniques (summarized in Table 3). Neumann et al. studied the interactions between the cerebellum, cerebral cortex, and the basal ganglia in patients with idiopathic dystonia. They used the technique of MEG recordings to investigate the cerebellum and the cerebral cortex with simultaneous, invasive local field potential recordings from the human pallidum by implanted deep brain stimulation electrodes. They found three brain networks associated with dystonia: (1) theta band coherence (4–8 Hz) from a pallido-temporal source, (2) alpha band coherence (7–13 Hz) from a pallido-cerebellar source, and (3) beta band coherence (13–30 Hz) from a cortico-pallidal source associated with sensorimotor areas. The directional coupling with the pallidal local field potentials was mainly in the theta and alpha band and for the MEG cortical source mainly beta band was observed. The pallido-cerebellar coupling was seen to be inversely correlated with dystonia severity. Based on the pallido-cerebellar connectivity findings, the involvement of the cerebellum in dystonia is evident [71]. In another coherence based study, Butz et al. used MEG and DICS analysis to investigate oscillatory coupling in patients with writer’s cramp, a form of focal dystonia. Six brain areas including ipsilateral cerebellum, contralateral thalamus, contralateral premotor and posterior parietal cortex, and contralateral and ipsilateral sensorimotor cortex were found to be involved when coherence was established during writing. In control subjects, the ipsilateral cerebellum and the contralateral posterior parietal cortex are primarily involved in writing, whereas in patients with writer’s cramp, sensorimotor cortices appeared to be additionally recruited. Thus, the abnormal interactions between the cerebellum and different parts of the cerebral cortex may associate with the pathophysiology of focal task related dystonia [72]. Furthermore, the importance of the cerebellum in dystonia was highlighted in a case study by Mahajan et al. In a 53-year-old patient with cervical dystonia, MEG recordings were performed before and after botulinum toxin injection and a sensory trick (yawning). Performing the sensory trick was associated with reduced beta frequencies and increased gamma frequencies in the parietal cortex. After botulinum injection, higher coherence activity was observed in the left temporal, parietal regions, and right and left regions of the cerebellum [73], indicating that the cerebellum may be involved in the improvement of dystonia in these maneuvers.

## Discussion

The overlapping natures of cerebellar dysfunction can be distinctly observed in the above described disorders of ataxia, tremor, and dystonia. Understanding cerebellar physiology is a step forward in better understanding the pathophysiology and mechanisms of movement disorders, and targeted neuromodulation in the cerebellum can thus be possible. This review focuses on studies that have used encephalographic techniques such as EEG and MEG in movement disorders with an emphasis on the cerebellum.

While human physiological studies do not have the same level of resolution as animal studies, animal models of ataxia, tremor, and dystonia provide other means to understand the role of the cerebellum in these movement disorders. To study ataxic disorders, animal models carrying various SCA genetic mutations have been created, which recapitulate key behavioral and pathological features of SCA patients [74–78]. In these animal models, abnormal Purkinje cell firing patterns have been identified, before eventual Purkinje cell loss. Specifically, slowed Purkinje cell firing activity, as well as irregular Purkinje cell firing, has been found to be the key physiological attributes for ataxia [79–81]. In studying parkinsonism, the 1-methyl-4-phenyl-1,2,3,6-tetrahydropyridine (MPTP) model used for electrophysiological recordings, helps mimic PD symptoms, which can further be used to assess oscillatory activity [82]. In addition, a mouse model with ET-like climbing fiber-Purkinje cell synaptic connections also developed synchronized and oscillatory cerebellar activity and action tremor [21], further supporting the role of oscillatory cerebellar activity in tremor, which has been observed in ET patients [62].

For dystonia, cerebellum-specific knockdown of DYT1, DYT11, and DYT12 in mice create burst firing frequency of Purkinje cells, which correlate with dystonic features in these animals [31, 83, 84]. Therefore, high frequency, burst firing patterns could be the cerebellar physiological underpinning for dystonia. Studies such as these are contributing factors in the advancement of cerebellar physiology.

Our review is centered on the cerebellar physiology in movement disorders. The studies outlined in this review help to advance the understanding of the physiological aspects of the cerebellum in movement disorders. These non-invasive techniques potentially can be implemented in clinical practice to evaluate ataxia, tremor, and dystonia. Even though EEG and MEG have limited spatial resolution, the high temporal resolution allows for these measures to evaluate physiological changes in real time. These studies demonstrate that recording of the oscillatory activity from the cerebellum is indeed possible and should be further applied to study a plethora of neurological disorders in the future. Note that there are studies using EEG and MEG to investigate physiological features, dynamics, and networks in brain regions, outside of the cerebellum in these movement disorders. We also identified these studies that have used common measures such as source localization, connectivity, and coherence in ataxia [85–91], tremor [92–129], and dystonia [130–152] to study brain physiology but these studies do not include the cerebellum; thus, they are not reviewed in detail here. The applications of EEG and MEG based source analysis and connectivity analysis have a wide range of utility. Not only can EEG and MEG be used to study movement disorders, but many studies have also utilized these tools to probe cognition. Therefore, these tools can also be used to study the non-motor symptoms of movement disorders in the future. Furthermore, EEG and MEG can be used to extract features in the spectral domain, which may serve as a biomarker to monitor therapeutic responses for ataxia, tremor, and dystonia. Additionally, degenerative ataxias, such as SCA, are progressive disorders, whether physiological recordings can be utilized as biomarkers to track disease progression deserve further investigation as part of the natural history study.

The studies included in this review with a focus on cerebellar physiology, mostly consist of network studies. Methods such as spectral analysis, source localization, and coherence analysis of the signals have been used in these studies. For ataxia, the cerebellar network and its connection with the frontal network appear to be important [45, 46]. The network studies for ET have shown the involvement of the cortico-brainstem-cerebello-thalamo-cortical loop playing a major role in tremor generation [53, 54]. Parkinsonian tremor has an involvement of the cerebello-diencephalic-cortical network [55, 58]. Finally, the cortico-pallidal connectivity linked with dystonic symptoms demonstrates the involvement of cerebellum [71], and furthermore, impaired cerebello-cortical networks have been observed in focal task related dystonia [72]. These network studies help us to understand the interactions between different brain regions in movement disorders. Manipulations of disease-related brain networks hold promise in therapeutic development in these disabling diseases. Also, EEG-based recordings consisting of evoked potentials (i.e., event related potentials) have been investigated in a handful of studies [47–50], which can potentially be developed into a physiological biomarker in the future. In addition, these studies demonstrate the possibility of using non-invasive techniques in exploring and advancing cerebellar physiology.

Additionally, EEG and MEG techniques have a potential application as a tool to guide neuromodulation in cerebellar disorders. Non-invasive neurostimulation techniques, such as transcranial direct current stimulation (tDCS) and TMS, have been used for cerebellar stimulation in movement disorders [153]. However, the effects of the stimulations are largely dependent on the neurological assessment, without a clear understanding of the underlying physiological attributes. Therefore, EEG and MEG can potentially record neural responses in the cerebellum in responses to neuromodulation [154, 155]. Therefore, studying cerebellar physiology using these techniques can advance the therapeutic development of neuromodulation for movement disorders.

Based on the studies described here, it is becoming clear that EEG and MEG are useful to investigate cerebellar physiology linked to ataxia, ET, parkinsonian tremor, and dystonia. In particular, EEG is easy to implement in clinical practice. These non-invasive physiological recordings further contribute to insights on cerebellar function and dysfunction, paving the way for better therapeutics for movement disorders. This review demonstrates that there are only few studies till now that focus on electrophysiological techniques to study the cerebellum in humans in the context of movement disorders; nonetheless, this unexplored area of neuroscience presents an exciting opportunity for research and therapeutic development.

## Figures and Tables

**Table 1:** Cerebellar physiological studies for ataxia

Authors	Techniques	Clinical syndrome	Number of patients	Number of controls	Methods	Findings/outcome
Naeije et al. [45]	MEG	Friedreich ataxia	18	-	Resting-state functional connectivity	Age of onset determines intrinsic functional brain architecture
Song et al. [46]	TMS-EEG	Multiple system atrophy-C	50	25	Connectivity analysis	Improvement of cerebello-frontal connectivity and balance functions
EEG studies on ataxia subjects without direct measurement of cerebellar physiology to investigate the cortical EEG changes in response to cerebellar degeneration						
Peterburs et al. [47]	EEG	SCA6, SCA 14,SAOA, ADCA III	16	16	Saccadic performance monitoring, event related potentials	In patients with cerebellar degeneration, performance monitoring is altered. Cerebellum plays a critical role in fast classification of saccadic accuracy
Li et al. [48]	EEG	SCA	12	12	Event related potentials, source localization	Cerebellum is essential for feed-forward control and feedback‐based control of speech production
Verleger et al. [49]	EEG	Cerebellar ataxia	12	10	Event related potentials, response force	The cerebellum as compared to the motor cortex is not mainly active in fine coordination but is mostly involvement in preparatory and executive activity
Yamaguchi et al. [50]	EEG	Ataxia-cerebellar degeneration	9	12	Event related potentials, reaction time	Limited contribution of the lateral cerebellum in visuospatial attention shift. Response related processing affected by cerebellar lesions
Visani et al. [51]	EEG	EPM1, SCA	11- EPM1, 11-SCA	11	ERD/ERS analysis	EPM1 patients-abnormal ERD/ERS dynamics, SCA patients- defective ERD lateralization
Aoh et al. [52]	EEG, EMG	SCA3	15	15	ERD/ERS analysis	Decreased ERS in patients with SCA3, mainly at frequencies 20–30 Hz

*MEG* magnetoencephalography, *EEG* electroencephalography, *EMG* electromyogram, *TMS* transcranial magnetic stimulation, *SCA* spinocerebellar ataxia, *SAOA* sporadic adult onset ataxia, *ADCA* autosomal dominant ataxia type III, *EPM1* progressive myoclonic epilepsy type 1, *ERD* event-related desynchronization, *ERS* event-related synchronization

**Table 2:** Cerebellar physiological studies for tremor

Authors and year	Techniques	Clinical syndrome	Number of patients	Number of controls	Methods	Findings/outcome
Muthuraman et al. [53]	EEG	ET	20	10	Coherence, source localization-DICS, connectivity-RPDC	Oscillating cerebral networks underlying classical ET and aging-related tremor differ, suggests a pathophysiological difference
Schnitzler et al. [54]	MEG, EMG	ET	8	-	Source localization- DICS, Partial coherence	Network of brain areas including the cerebellum shows oscillatory interactions, leading to a rhythmic modulation of muscle activity becoming apparent as tremor
Timmermann et al. [55]	MEG, EMG	PD resting tremor	6	-	Source localization- DICS, coherence	Tremor-related oscillatory activity within a cerebral network, with abnormal coupling in a cerebello-diencephalic cortical loop and cortical motor and sensory areas contralateral to the tremor hand
Pollock et al. [56]	MEG, EMG	PD	10	11	Source localization- DICS, coherence	Decreases in cerebro-cerebral coupling between thalamus and motor cortical areas after levodopa intake. Tremor oscillatory networks consists of sources in contralateral primary sensorimotor cortex, supplementary motor area, contralateral premotor cortex, thalamus, secondary somatosensory cortex, posterior parietal cortex and ipsilateral cerebellum
Muthuraman et al. [58]	EEG-EMG	ET, PD	ET-34, PD-34	34	Coherence, Source localization- DICS, connectivity-TPDC	Differing topography of cerebellar activity in PD, ET and mimicked tremor
Pedrosa et al. [59]	EEG, MRI	ET	20	20	Source localization- DICS	Increases within frontal motor activity are associated with tremor emergence, and tremor amplitude reduction is linked to changes in cerebellar activity
Muthuraman et al. [57]	EEG, EMG	PD, ET	PD-10, ET-10	10	Source localization- DICS, RPDC	Voluntary tremor has a unidirectional flow from the diencephalon to cortex. Explanations about pathological and voluntary tremors having similar neuronal pathways, but pathologic tremors are unable to be controlled voluntarily. Cerebellar source found in ET, PD patients and healthy subjects
Muthuraman et al. [60]	EEG, EMG	Orthostatic tremor	15	-	Source localization- DICS, coherence	Initial bilateral corticomuscular coherence pattern in orthostatic tremor, followed by a segregated unilateral pattern. Standing tremor frequency consists of sources in the primary motor leg area, supplementary motor area, primary sensory cortex, prefrontal/premotor cortex, thalamus, and cerebellum
Südmeyer et al. [61]	MEG, EMG	Postural tremor-WD	5	-	Source localization- DICS, coherence	Synchronized network consisting of the cerebello–thalamo–cortical network, involving primary sensorimotor cortex, supplementary motor area, premotor cortex, and posterior parietal cortex generates WD postural tremor
Pan et al. [62]	Cb-EEG	ET	20	20	In vivo analysis, Time frequency analysis	Excessive cerebellar oscillations in patients with ET
Wong et al. [67]	Cb-EEG	Familial ET, Sporadic ET	Familial-22, Sporadic-18	20	Spectral analysis	In Familial ET cerebellar oscillations are associated with tremor severity but not sporadic ET. Conserved cerebellar oscillations in familial and sporadic ET patients
Bosch et al. [68]	EEG	PD	75	39	Spectral analysis	Resting state, theta band (4–7 Hz) cerebellar oscillations are abnormal
Bosch et al. [69]	EEG	PD	10 PD- with PI, 11 PD- without PI	15	Spectral analysis	Association of mid-frontal and mid cerebellar regions in postural stability and theta frequency oscillations (4–7 Hz) are important for postural control in PD
Bosch et al. [70]	EEG	PD	79	37	Time frequency analysis, connectivity analysis	Altered cerebellar oscillations in motor and cognitive task performances in PD

*EEG* electroencephalography, *MEG* magnetoencephalography, *EMG* electromyogram, *Cb-EEG* cerebellar EEG, *MRI* magnetic resonance imaging, *ET* essential tremor, *PD* Parkinson’s disease, *WD* Wilson’s disease, *DICS* dynamic imaging of coherent sources, *TPDC* temporal partial directed coherence, *RPDC* renormalized partial directed coherence, *PI* postural instability

**Table 3:** Cerebellar physiological studies for dystonia

Authors and year	Techniques	Clinical syndrome	Number of patients	Number of controls	Methods	Findings/outcome
Neumann et al. [71]	DBS electrode, MEG	Generalized, segmental, cervical dystonia	9	-	Coherence, Granger-based directionality analysis	Three spatially and spectrally distinct, interconnected cortico-pallidal circuits, including one pallido-cerebellar circuit
Butz et al. [72]	MEG, EMG	Dystonia, writer’s cramp	8	11	Source localization-DICS, coherence	Strong coherence between both primary sensorimotor cortices, reduced coherence between cerebellum and posterior parietal cortex
Mahajan et al. [73]	MEG	Cervical dystonia	1	-	Pre- and post-botulinum toxin injection and sensory trick, Time–frequency analysis, coherence	Increased gamma frequency may associate with increased GABAergic activity. The increased connectivity in the right cerebellar region depicts the role of cerebellum in dystonia pathogenesis is

From: Physiological Recordings of the Cerebellum in Movement Disorders

*DBS* deep brain stimulation, *MEG* magnetoencephalography, *EMG* electromyogram, *DICS* dynamic imaging of coherent sources
